# Improving Reliability of 1 Selector-1 ReRAM Crossbar Arrays Through Hybrid Switching Methods

**DOI:** 10.3390/ma18040761

**Published:** 2025-02-09

**Authors:** Hyun Kyu Seo, Min Kyu Yang

**Affiliations:** Department of Artificial Intelligence Convergence, Sahmyook University, Seoul 01795, Republic of Korea; seohyunkyu0811@gmail.com

**Keywords:** resistive random-access memory, selector, crossbar array

## Abstract

In this study, an innovative switching approach is explored to improve the reliability of 1 Selector-1 ReRAM (1S1R) devices, integrated into a 4K crossbar array (CBA). The key innovation is the use of DC sweeping for set operations and AC single-pulse resetting to minimize device stress and prevent breakdown. The selector, based on a GeSeTe ovonic threshold switching (OTS) element, demonstrated excellent endurance (>10^12^ cycles), fast switching (<100 ns), and high device-to-device uniformity (<5% variability). The ReRAM, constructed with Pt/LiNbO_x_/W, exhibited robust bipolar resistive switching, multi-bit capability, and endurance exceeding 10^12^ cycles. The integrated 1S1R CBA demonstrated reliable retention and low variability in operation, showing potential for high-performance, high-density memory applications.

## 1. Introduction

The increasing demand for high-density and energy-efficient memory solutions has positioned crossbar arrays (CBAs) as a promising architecture for next-generation memory devices. CBAs offer high integration density and scalability; however, challenges such as sneak currents and device reliability continue to hinder their widespread adoption. Sneak currents, which occur due to unintended current paths in unselected cells, degrade read/write accuracy and increase power consumption. Device reliability is also a significant concern, as repeated switching cycles can lead to material degradation and performance variability [1,2,3].

To address these challenges, the integration of a selector with resistive random-access memory (ReRAM) in a 1 Selector-1 ReRAM (1S1R) configuration has emerged as an effective solution. This configuration enhances read/write accuracy and minimizes sneak path currents by providing an inherent non-linearity in the current-voltage characteristics of the device. A typical 1S1R structure consists of an ovonic threshold switching (OTS) selector and a ReRAM cell. The OTS selector functions as a threshold-dependent switch that remains in a high-resistance state until a critical voltage is applied, effectively blocking leakage currents in unselected cells and preserving data integrity [4,5,6,7,8,9,10,11,12,13,14,15]. Meanwhile, the ReRAM cell provides reliable resistive switching behavior, enabling non-volatile data storage through controlled filament formation and rupture mechanisms [16,17,18,19,20,21,22,23,24,25,26,27,28,29,30,31]. Recent advances in material engineering and device structuring have further improved the scalability and performance of 1S1R arrays, making them more suitable for commercial applications [32,33,34].

Despite these advances, conventional 1S1R devices still face challenges, primarily due to the repeated application of high voltages during both set and reset operations. This continuous stress can accelerate material degradation, leading to potential breakdown and increased variability in device performance [35,36,37]. These reliability concerns become more pronounced in large-scale arrays, where consistent and uniform operation across multiple cells is critical [38,39,40,41,42]. Recent studies have proposed hybrid switching techniques to mitigate these issues, incorporating optimized pulse schemes to balance performance and endurance.

In this study, we propose a novel hybrid switching approach that employs DC sweeping for the set operation and an AC single-pulse for the reset process. The proposed method significantly reduces electrical stress during the reset phase, improving the device’s endurance and reliability. The effectiveness of this approach is validated using a GeSeTe-based OTS selector and a LiNbO_x_-based ReRAM, integrated into a 4K CBA. Our results demonstrate the potential of this hybrid switching technique for achieving high-performance and scalable memory applications, contributing to the advancement of next-generation non-volatile memory technologies.

## 2. Materials and Methods

### 2.1. Device Fabrication

The fabrication of the OTS device began with the deposition of a 30 nm tungsten (W) layer onto a Si/SiO_2_ substrate using physical vapor deposition (PVD). The bottom electrode (BE) pattern of the two-terminal unit cell was defined through photolithography and dry etching. Dry etching was performed using inductively coupled plasma reactive-ion etching (ICP-RIE) with an ICP source power of 300 W and a substrate bias power of 50 W. The etching gases included 4 sccm of argon (Ar) and 30 sccm of chlorine (Cl_2_), maintained at a process pressure of 5 mTorr. The etching rate was approximately 1 nm/min. After etching, residual photoresist (PR) was cleaned using acetone, isopropyl alcohol, and deionized (DI) water. A GeSeTe layer was then deposited using sputtering at 40 W radio frequency (RF) power, with a base pressure of 5 × 10^−7^ Torr and a working pressure of 2.8 mTorr. The sputtering process employed an Ar gas flow rate of 15 sccm. Finally, a 50 nm W top electrode (TE) was deposited through direct current (DC) magnetron sputtering after defining the TE pattern using photolithography and lift-off techniques. The DC sputtering process was performed at a base pressure of 1 × 10^−6^ Torr, a working pressure of 2.8 mTorr, and a DC power of 80 W with an Ar flow rate of 15 sccm. For the ReRAM device, a 100 nm platinum (Pt) layer was deposited on a Si/SiO_2_ substrate via PVD. The Pt bottom electrode patterning and etching processes followed the same procedure used for the OTS device with ICP source power of 350 W and a substrate bias power of 150 W. The etching gases included 4 sccm of Ar and 30 sccm of Cl_2_, maintained at a process of 3 mTorr. The etching rate was approximately 1 nm/min. After etching, residual PR was cleaned using acetone, isopropyl alcohol, and DI water. Subsequently, a TaO_x_ switching layer was deposited by sputtering at 80 W RF power, with a base pressure of 5 × 10^−7^ Torr, a working pressure of 5 mTorr, an Ar flow rate of 20 sccm, and an oxygen (O_2_) flow rate of 15 sccm. Finally, a 50 nm W TE was deposited using the same method as employed for the OTS device. The 1S-1R structure was fabricated using a mask pattern different from the single-structure mask pattern described above, with the W TE of the fabricated ReRAM device simultaneously serving as the BE for the OTS selector. The OTS selector and the TE were deposited using the same methods as described above. This stepwise integration process ensured strong electrical contact and precise alignment between the OTS selector and the ReRAM device. The combined structure leveraged the OTS selector’s threshold switching capabilities and the ReRAM’s resistive switching characteristics, enabling efficient performance and scalability for high-density memory applications.

### 2.2. Electrical Measurements

Electrical characteristics of individual devices and the integrated 1S1R were assessed using a semiconductor parameter analyzer (SPA, Keithley 4200 SCS; Beaverton, OR, USA). During AC-based pulse operation, an arbitrary function generator (AFG, Agilent 81110A; Beaverton, OR, USA) and an RF electric-circuit switch box were used. The electrical pulses were generated by the AFG, and the conductance states were verified by the SPA. Most electrical measurements were executed at room temperature (25 °C), but the retention measurement was conducted at both room temperature and 85 °C. A hot stage using a temperature controller was employed to regulate the measurement temperature. During the synaptic operation of the ReRAM, the incremental step pulse programming (ISPP) method was adopted, in which incrementally increasing electrical pulses were applied to the device. Using this method, the ReRAM was potentiated or depressed to mimic the neuronal synaptic operation of the human brain. After applying the electrical pulse, a verifying step followed to obtain the conductance states of the device using SPA. All electrical measurements were performed using a LabVIEW™-based control program.

## 3. Results and Discussion

### 3.1. Characteristics of the ReRAM and OTS Devices

**Figure 1:**
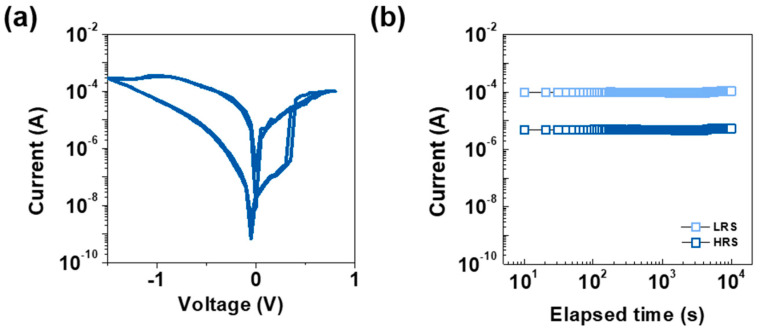
(**a**) DC I−V characteristics showing bidirectional resistive switching device behavior. (**b**) Retention performance demonstrating stable current levels for both HRS and LRS.

**Figure 2:**
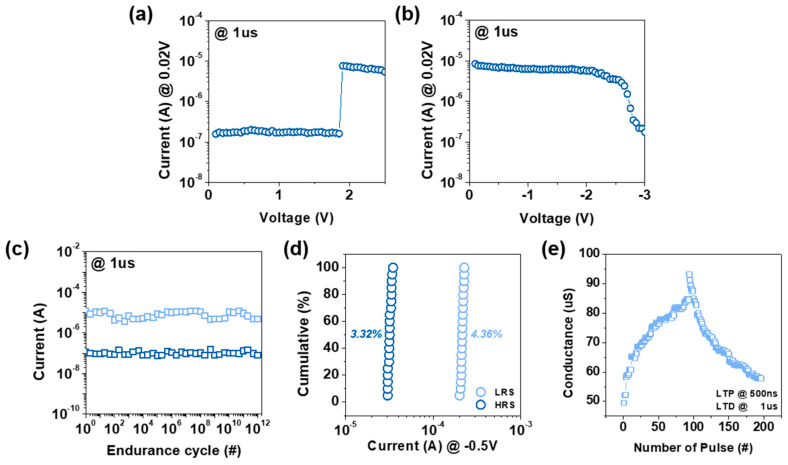
(**a**) Set operation characteristics and (**b**) reset operation characteristics of the Pt/LiNbO_x_/W device. (**c**) Endurance performance over multiple cycles. (**d**) Cumulative current distribution across multiple cells. (**e**) Synaptic behavior with stable conductance modulation.

Figure 1 illustrates the electrical characteristics and retention properties of the Pt/LiNbO_x_/W-based ReRAM device. Figure 1a shows the DC I-V characteristics, demonstrating the bidirectional resistive switching behavior. The device transitions from the high-resistance state (HRS) to the low-resistance state (LRS) at a threshold voltage (V_th_) of approximately ±0.3 V within a sweep voltage range of +0.8 V to −1.5 V. The sharp switching and low off-current observed confirm reliable resistive switching with minimal leakage. Figure 1b presents the retention characteristics of the device, measured at a read voltage (V_read_) of −0.5 V. The current remains stable for both HRS and LRS over 10^4^ s, indicating excellent data retention performance. These results confirm the Pt/LiNbO_x_/W-based ReRAM device’s suitability for non-volatile memory applications, with robust switching and reliable data storage capability. Figure 2 demonstrates the electrical performance and reliability of the Pt/LiNbO_x_/W-based ReRAM device.

In Figure 2a, the set switching behavior is shown with the device transitioning from the HRS to the LRS at approximately 1.8 V within 1 µs at a V_read_ of 0.02 V. Figure 2b highlights the reset switching, where the device returns from LRS to HRS at approximately −3 V within 1 µs. The endurance characteristics presented in Figure 2c show stable current levels for both HRS and LRS over 10^12^ cycles, confirming the device’s excellent reliability and robust cycling performance. Figure 2d shows the cumulative current distribution of 20 cells measured at −0.5 V, with a distribution ratio of 3.32% for HRS and 4.36% for LRS, indicating consistent and uniform switching behavior across the cells. Lastly, Figure 2e highlights the long-term potentiation (LTP) and long-term depression (LTD) characteristics under incremental step pulse programming (ISPP), where stable conductance modulation is observed over 200 pulses with pulse widths of 500 ns for LTP and 1 µs for LTD. These results demonstrate the high endurance, reliable switching characteristics, and synaptic behavior of the Pt/LiNbO_x_/W-based ReRAM device, making it a promising candidate for high-performance non-volatile memory and neuromorphic computing applications.

**Figure 3:**
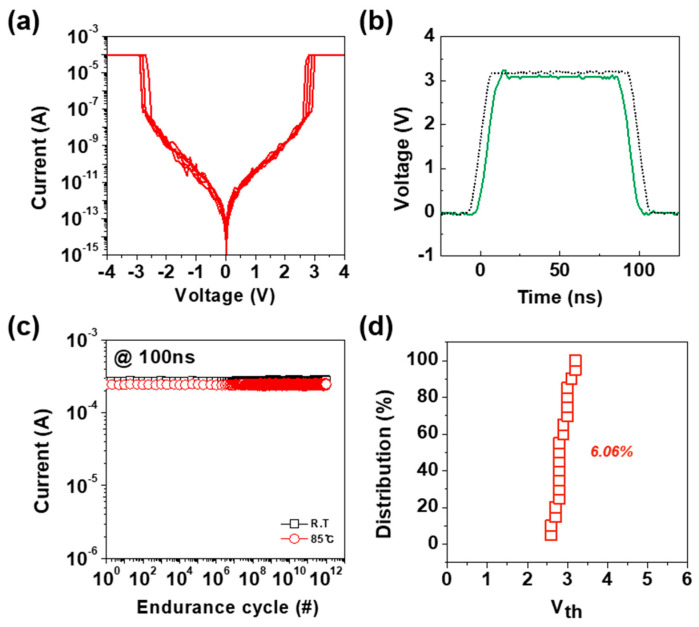
(**a**) DC I−V characteristics showing bidirectional threshold switching behavior. (**b**) Voltage waveform used to evaluate switching dynamics. (**c**) Endurance performance over multiple cycles. (**d**) Statistical distribution of threshold voltage.

Figure 3a illustrates the DC I-V characteristics of the W/GeSeTe/W device, measured over an operating voltage of −4 V to 4 V. The graph demonstrates the bidirectional threshold switching behavior, with the V_th_ observed at approximately ±2.7 V. The current remains in the off-state (I_off_) with a leakage current of approximately 400 pA until the applied voltage reaches V_th_, beyond which a rapid increase in current occurs, indicating the transition from HRS to LRS. Upon reducing the voltage, the device returns to its HRS, confirming the reversible switching behavior. Figure 3b presents the time-resolved voltage waveform applied to the OTS device during the switching operation. The applied waveform consists of a voltage pulse with a specific amplitude and duration, designed to induce the threshold switching behavior of the device. The graph demonstrates the voltage increase, stabilization at the peak level, and subsequent decrease over the course of the measurement. The application of this voltage pulse is crucial for analyzing the switching dynamics of the OTS device, as it provides insights into key performance parameters such as response time, threshold voltage stability, and write latency. The flat region at the peak voltage indicates the duration for which the applied voltage exceeds the V_th_, allowing precise measurement of the time required for the device to transition from the HRS to the LRS. This transition time is a critical metric for evaluating the device’s suitability for high-speed memory applications. Moreover, the voltage waveform enables the assessment of switching consistency across multiple cycles, helping to identify potential variability and drift over prolonged operation. The ability to precisely control and optimize the switching time is essential for ensuring reliable and energy-efficient operation of selector devices in CBAs. Achieving minimal switching delay not only enhances the overall memory performance but also reduces power consumption, making the device suitable for high-density applications [43,44,45]. Additionally, understanding the temporal characteristics of the OTS device is critical for mitigating potential crosstalk or interference with neighboring memory cells, which is a common challenge in densely integrated memory arrays. By optimizing the applied waveform parameters, it is possible to improve the scalability and operational reliability of next-generation non-volatile memory technologies. These improvements and insights provided by the applied voltage waveform, as illustrated in Figure 3b, offer valuable guidance for optimizing the OTS device’s performance under various operating conditions and ensuring its practical application in advanced memory architectures. Figure 3c shows the endurance performance of the OTS device under 100 ns pulse operation at room temperature (RT) and elevated temperature (85 °C). The current remains stable over 10^12^ switching cycles, demonstrating excellent endurance and thermal stability. The minimal deviation in the current under high-temperature conditions indicates the robustness of the OTS device for operation in challenging environments. High endurance and thermal stability are essential for selector devices in high-density memory applications, as they ensure long-term reliability and performance under continuous stress. Furthermore, prolonged operation at high temperatures can induce atomic rearrangement within the OTS material, potentially affecting the stability of conduction pathways and the repeatability of switching characteristics. In particular, GeSeTe-based OTS materials are susceptible to phase transitions at elevated temperatures, which may lead to performance degradation. Therefore, the high-temperature endurance performance demonstrated in Figure 3c is significant, as it confirms the stability and reliability of the OTS device under conditions that could otherwise pose challenges to its operation [46,47]. These results confirm that the OTS device can withstand prolonged cycling without significant degradation, making it a strong candidate for use in next-generation memory technologies. Figure 3d presents the statistical distribution of the V_th_ for the OTS device, measured across 20 individual cells. The x-axis represents the V_th_, while the y-axis shows the cumulative percentage distribution of the V_th_ values. The narrow spread of the V_th_ values, quantified by the standard deviation (6.06%), demonstrates the excellent uniformity and reliability of the device’s switching characteristics across multiple cells. This analysis is critical in the context of selector devices for high-density memory arrays, where consistent and predictable switching behavior across a large number of cells is essential for ensuring robust device operation. A narrow distribution of V_th_ values minimizes the variability, reducing the risk of unwanted interference or misoperation in crossbar architectures. Furthermore, the small variation in V_th_ indicates the stable material properties and device performance over repeated operations, even when measured across multiple cells. This consistency ensures long-term reliability in non-volatile memory applications. The observed uniformity in V_th_ distribution across the 20 cells highlights the suitability of the OTS device for use as a selector in memory arrays. Such uniformity enables the precise control of memory cell activation while suppressing sneak current paths, making it a strong candidate for achieving high yield and scalability in next-generation memory technologies.

### 3.2. Integration and Performance of the 1S1R Device

**Figure 4:**
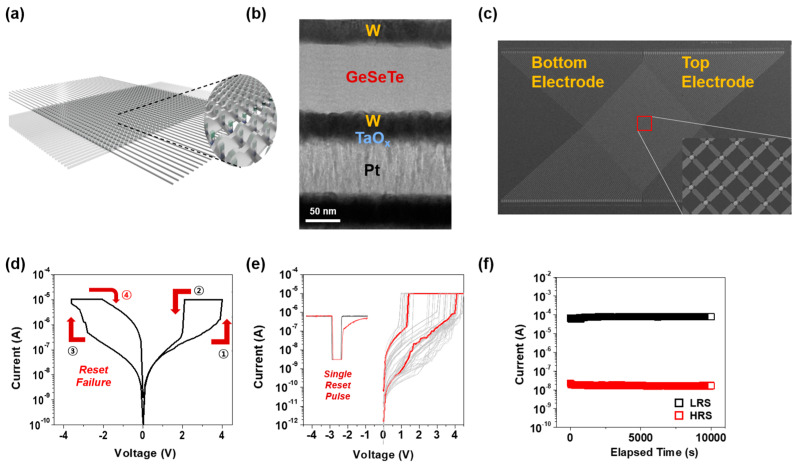
(**a**) Schematic of the 1S1R CBA. (**b**) TEM image of the 1S1R structure. (**c**) SEM top−view image of the fabricated 4k CBA. (**d**) DC I−V characteristics showing reset failure. (**e**) Stable operation achieved through single reset pulse. (**f**) Retention performance of the 1S1R device.

Figure 4 illustrates the design, structural characterization, and electrical performance of the integrated 1S1R device implemented in a 4K CBA to enhance reliability through hybrid switching methods. Figure 4a presents a schematic of the 1S1R CBA, demonstrating its potential for high-density memory integration. Figure 4b shows the TEM (JEOL, Akishima, Japan) image of the 1S1R device structure, consisting of W/GeSeTe/W as the selector and Pt/TaO_x_/W as the memory element. The SEM (ZEISS, Jena, Germany) top-view image of the fabricated 4K CBA, displayed in Figure 4c, confirms the successful integration of the 1S1R architecture. Figure 4d shows the DC I-V characteristics of the 1S1R device with a compliance current (CC) of 1E-5 A. When operating under a bipolar DC sweep, the device transitions from HRS to LRS at point 1, and successfully returns to HRS at point 2 near 1.5 V. However, in the negative voltage region, although the device transitions to LRS at point 3, it fails to return to HRS at point 4, leading to performance degradation in subsequent cycles. This reset failure in the negative region hinders reliable device operation. To address this issue, a single reset pulse with a width of 1 µs and an amplitude of −5 V was applied, as shown in Figure 4e, successfully mitigating the reset failure and ensuring stable operation. This transition to single-pulse resetting represents a critical improvement in device reliability by enabling consistent switching performance. The hybrid switching approach, where the positive region sweep is maintained while employing a negative pulse for reset, prevents incomplete transitions and enhances device endurance. The negative pulse ensures that the device fully resets from LRS to HRS, thus improving overall reliability and preventing cumulative degradation. Finally, Figure 4f demonstrates the retention characteristics of the 1S1R device, measured at a read voltage of 2 V, where stable current levels for both LRS and HRS are maintained over 10,000 s. These results highlight the effectiveness of the hybrid switching method in improving the reliability of 1S1R CBAs, aligning with the focus in this study on optimizing reset operations through the integration of DC sweeps and single-pulse techniques.

## 4. Conclusions

In this work, a reliable 1S1R memory device integrated into a 4K CBA is demonstrated. The proposed novel switching scheme (DC sweep for setting and AC pulse for resetting) effectively reduces the device stress and prevents breakdown compared to conventional DC-only methods. By minimizing cumulative electrical stress during reset operations, this approach extends device endurance and operational lifespan, which is a critical improvement for large-scale memory applications. The integration of a GeSeTe-based selector and LiNbO_x_-based ReRAM provides a synergistic effect, achieving robust endurance, excellent retention, and low variability. Compared to traditional 1S1R architectures, the proposed hybrid switching method enables faster switching speeds and reduced power consumption, making it more suitable for next-generation high-density memory solutions. Furthermore, the reduction in reset failure rates and the improved uniformity across large memory arrays highlight the effectiveness of the hybrid switching scheme in addressing scalability challenges. These findings underscore the potential of 1S1R architectures with hybrid switching methods for next-generation memory technologies, offering a promising path toward efficient, reliable, and high-performance data storage solutions. Future works will focus on further optimizing the switching conditions and exploring the applicability of the proposed technique to even larger memory arrays and different material systems.

## Data Availability

The original contributions presented in this study are included in the article. Further inquiries can be directed to the corresponding author.
